# Discontinued General Anesthesia

**Published:** 1918-12

**Authors:** 


**Discontinued General Anesthesia.** By H. CHAPUT. *Bulletin de la Société de Chirurgie*, June 11, 1918.

Discontinued anesthesia is a process of general anesthesia to which the author has already called attention. The essential characteristics are :

1. Administration of minima doses of anesthetic agent.
2. Renewal of anesthesia as soon as sensitiveness or motility reappear enough to hinder the operation.

Chaput gives anesthesia only when the operatory region has been disinfected and sterilized. Inhalations are given and stopped until the patient shows signs of restlessness, then renewed, and so on.

Discontinued anesthesia consists, therefore, in a series of loss of consciousness, alternated with reappearance of motor reaction sufficient to hinder the operator. It is very similar to that used in childbirth, though the technic of the latter has never been properly defined. As the author remarks, discontinued anesthesia is not a new method of his invention. Generally patients lose consciousness, but, in some cases, though they do not feel the pain, they can answer questions.

During discontinued anesthesia the cornean reflex is preserved; the face is not discolored; the pupils are hardly modified; the pulse is strong and beating well; there is no vomiting after the operation; nor is there any cardiac or respiratory syncope, provided, however, there is no deglutition of the tongue. The after effects are very mild indeed. In cases of sus-ombilical operations the patients can even get up the same day.

Discontinued anesthesia can be combined with local or lumbar anesthesia, allowing the surgeon to begin the operation without any of the terror apprehensions on the part of the patient.

A fact to be taken into account is that alcoholic subjects require a stronger dose than patients not taking exciting drinks.

Owing to this method chances of pulmonary congestion are greatly reduced, so is also the alteration of the visceral parenchymas. So far Chaput has practised more than a hundred of these discontinued anesthesias.

There are a few objections to this method.

1. It is a hindrance to the surgeon, which is remedied easily.

2. It has been said that patients were not properly anesthetized and suffered, but all of them, on being asked, declared they had felt nothing at all.

3. The majority of surgeons believe it dangerous, but, on the contrary, the risks of syncope are infinitely less than in complete anesthesia.

4. Discontinued anesthesia is insufficient, it is true, for operations which demand entire muscular resolution, such as lithotrity, abdomen operations and others equally grave.

At any rate discontinued anesthesia is neither as dangerous nor as tiring as complete anesthesia, and it spares the viscera in a large degree.

Dr. Morestin agrees with this method, which he practises a great deal at the St. Louis hospital, for cancers of the face or tumors of the upper region. For old people this practice is recommended, since they offer a weak resistance and are so exposed to pulmonary complications.

Morestin also thinks that making the patient count aloud is an excellent means of finding out the progress of obnubilation. In the course of the operation, when tumultuous reactions take place, there is an excellent way of coping with them by practising a little bleeding on a vein near the operating center about 50 to 100 grammes. The result is simply astonishing. Many students or doctors have since recognized what a useful resource it is.

